# An Opinion on How Nanobiotechnology is Assisting Humankind to Overcome the Coronavirus Disease 2019

**DOI:** 10.3389/fbioe.2022.916165

**Published:** 2022-06-13

**Authors:** Tania Limongi, Francesca Susa

**Affiliations:** Department of Applied Science and Technology, Politecnico di Torino, Turin, Italy

**Keywords:** COVID-19, SARS-CoV-2, nanobiotechnology, health emergencies, prevention, diagnostics, treatments, patents

## Introduction

Analyzing results from a PubMed search done at the end of March 2022, more than 550,000 scientific publications relating to “Biotechnology” have been published. At the same time, if searching for “Nanobiotechnology,” it can be seen that more than 7,100 publications have been written in the last 20 years, with an ever-increasing annual publication rate. Nanobiotechnology, a relatively young branch of knowledge, is the crossing point between bio and nano technology, and comprises a wide range of nanotechnological applications in the life sciences field. In the last 2 years, just one hundred years after the last Pandemic Flu, humanity has sadly been hit by one of the worst infectious disease emergences of modern times. We believe that only in conjunction with this terrible health crisis have the real power of what are commonly recognized as Nanobiotechnologies been realized. Since the onset of the pandemic, these technologies have been the subject of heated discussions for scientists, clinicians, journalists, politicians, and ordinary people. For instance, next to the most accredited zoonotic transfer origin of SARS-CoV-2 (Andersen et al., 2020), it was even speculated (Casadevall et al., 2021) to have a nanobiotechnological origin in one of the biosafety level-4 biological laboratories of the Wuhan Institute of Virology.

Although there is still no way to reach unquestionable conclusions on the origins of this pandemic, allowing us to establish how much was due to chance, nature, or to the fast development of human technologies, in this opinion we will prove how much nanobiotechnology is doing in assisting the whole scientific community in quickly and effectively resolving this global health crisis. We want to highlight how much nanobiotechnology is contributing in such a short time with nanomaterials and technology to the development of safe and efficient solutions to prevent, diagnose, and treat COVID-19 and any infections of the same nature.

## Why and How to Use “Nano” to Assist “Bio”

For at least a couple of decades, technology and nanoscience have demonstrated an important role in the design of effective solutions for biomedical applications, offering new methods and materials. Due to their dimensions ranging from a few to hundreds of nanometers, nanomaterials, whether of biological, inorganic, or synthetic origin, demonstrate exclusive performances (Xiao et al., 2022) as core elements or components of a wide range of nanotechnological solutions to various kinds of diseases.

Nanobioengineered solutions, including nanomaterials and nanovehicles, are ever more frequently used for medical microbiology, tissue repair and regeneration, drug delivery, diagnosis, and treatment of inflammatory and cancer diseases (Susa et al., 2019; Contera et al., 2020). Furthermore, a wide range of micro and nanofabrication processes allows the design and fabrication of highly sensitive sensors and transistor-based biosensors able to detect and analyze multicomponent mixtures from human samples for early diagnosis or real-time continuous monitoring (Coluccio et al., 2015; Nagamine and Tokito, 2021; Park et al., 2021; Sung et al., 2021; Finnegan et al., 2022; Kaloumenou et al., 2022).

The impact of the use of nanotechnologies and of their related and waste products must be assessed based on both people and the environment. In agreement with most of our colleagues, we believe that nanobiotechnology-based solutions deserve special administrative rules (Osman, 2019; Allan et al., 2021; Carlander and Skentelbery, 2021). Although regulatory organizations, such as the Global Coalition for Regulatory Science Research, the European Commission’s Joint Research Centre, the United States Environmental Protection Agency and the U.S. Food and Drug Administration, have begun to regulate the potential problems arising from the use of nanotechnologies, there is still an urgent need to define universally shared toxicity standards and updated regulatory guidelines (Subhan and Subhan, 2022).

## Has the Nanotechnological Contribution Made a Difference in the Fight Against COVID-19 Compared to Other Epidemics?

Among the various pathologies, infectious ones involve a very high incidence rate and, obviously, actions against these diseases, such as human immunodeficiency virus, malaria, and tuberculosis, are particularly challenging, considering their unending spread and high mortality rate (Kirtane et al., 2021).

More than 2 years ago, humanity was confronted by the SARS-CoV-2 virus that caused COVID-19 disease. There is no question that this tragic event has radically changed the lives of billions of people, since it rapidly spread everywhere, leading to the closure of public and private facilities, filling hospitals, and shutting down worldwide economies and social activities. Professionals from different backgrounds joined forces to try to stem this pandemic with all the losses in human and socio-economic life that it entailed. The massive investigation effort made for COVID-19 prevention, diagnosis, and treatment started 2 years ago, is still ongoing, and should be recognized as a prodigious success since it is substantially contributing to this pandemic disease becoming endemic.

Many solutions, such as vaccine production and medication repurposing, have been adopted and, among the various strategies used, nanotechnology undoubtedly has provided better solutions in the implementation of valid preventive and diagnostic actions, such as through the design of faster and more effective treatments (Weiss et al., 2020).

A very recent (March 2022) query, done in the Web of Science Core Collection, for the string “COVID-19 nanotechnology” provided more than 400 papers published between 2020 and 2022. By refining the search, adding respectively the words “prevention,” “diagnostics,” and “treatments,” we noticed that about 10% concern prevention application, 40% diagnostics, and 50% treatments. This bibliographic research had clearly shown how, in just 2 years, a remarkable variety of nanotechnological approaches assisted scientific and medical communities in developing nanomaterials for the implementation of disinfection systems, molecular or antigenic diagnosis kits, antiviral drugs, and nanoarchitectured vaccines (Ruiz-Hitzky et al., 2020).

## Have Patents, During the COVID-19 Emergency, Promoted DISCLOSURE OF INVENTIONS, REDUCING DUPLICATION OF RESEARCH, COSTS OF RESEARCh, and bargaining?

Given the exceptional number of scientific publications produced on the just introduced topic by colleagues in these pandemic years, in this opinion article for the Nanobiotechnology section of the journal Frontiers in Bioengineering and Biotechnology, we focused on one of the most important engineering and technological aspects of the discussion, patents.

One of the first works on this aspect of the pandemic is the one in which Ruiz-Hitzky and others provided an indication on patented work related to nanotechnology applied to coronaviruses (Ruiz-Hitzky et al., 2020). They carried out a systematic search on the distribution of patents dealing with SARS-CoV viruses (comprising SARS-CoV and SARS-CoV-2) by using the “coronavirus and nano” topics to refine the query in the ESPACENET patent website. Since their article was published in September 2020 and their ESPACENET database query focused generically on the terms “coronavirus and nano,” on April 2022, we did a new search on both VaxPal and ESPACENET websites, looking for patents related to the applications of nanobiotechnologies for COVID-19 prevention, vaccines, diagnosis, and treatments. The results confirmed that, in all the application areas of our interest such as prevention, diagnostic, and therapeutic, nanotechnologies have given and are greatly assisting in the successful management of a sudden and unexpected global emergency like the COVID-19 pandemic. More in detail, as summarized in Table 1, considering the 45 resulting patents, four refer to prevention, 20 to vaccines development, 14 to diagnostics, and seven to therapeutic applications. Most of them were filed by companies and academic institutes (18 and 16 patents, respectively), eight by private organizations, and three by governmental entities.

**TABLE 1:** Patents related to the applications of nanobiotechnologies for COVID-19 prevention, vaccines, diagnosis, and treatments. Data from Tavares et al. (2022) and from a query on VaxPal and ESPACENET websites carried out on April 2022.

Application	Nanotechnology	Product	Patent number	Producer
Prevention	Porous structure	Filtration article for personal protective equipment	US2021331107A1	Company: Ion Defense Tech LLC
	Nanoparticles	Compositions and methods for reducing the transmissivity of illnesses using an oral delivery system	US2022080020A1	Private : Montagnier Luc and Lorenzen Lee H
	Lipid nanoparticles	Topical application of povidone-iodine on mucous membranes	WO2020232515	Private: Firebrick Pharma PTY LTD
	Silver nanoparticles	Nanosilver spray	CN111466407	Private: Dong Nan
Vaccine	Lipid nanoparticles	mRNA-1273	US10898574B2	Company: ModernaTX
		Tozinameran (BNT162b2)	WO2017075531A1	Company: Pfizer-BioNTech: Acuitas
		Zorecimeran (CVnCoV)	WO2021123332A1	Company: CureVac
		ARCoV	CN111333704A	Company -Government: Academy of Military Science, Walvax Biotechnology, Suzhou Abogen Biosciences
		LNP-nCoVsaRNA	WO2021212101A1	Academic: Imperial College of London
		Vaccine	KR2020118386	Private: Lee Ho Jun
			CN112662695	Academic: Institute Medical Biology CAMS
			IN202021024459	Private: Lohagaonkar Kunal Sambhaji, Lohagaonkar Manisha Kunal
			CN111218458	Company: Zhuhai Lifanda Biotechnology CO LTD
			CN112300251	Academic: Sichuan University
	Proteo-lipid vehicle	Covigenix VAX-001	US2019367566A1	Company: Entos Pharmaceuticals
	Nanoparticles	NVX-CoV2373	WO2021154812A1	Company: Novavax
		Nano-vaccine	CN111607002	Academic: Sun Yat Sen University
		Vaccine	CN112480217	Company: Guangzhou Ribobio CO LTD, Agena Biopharmaceutical CO LTD
	Ferritin nanoparticles	SpFN	WO2021178971A1	Government: Walter Reed Army Institute of Research
	Gold nanoparticles	Vaccine	RO134811A0	Academic: Institutul Nat De Cercetare Dezvoltare Pentru Fizica Tehnica IFT Iasi
	Aluminum nanoparticles	Vaccine	CN111939253	Company: Hefei Novel Gene Tech Service CO LTD
	Self-assembling nanoparticles	Vaccine	US20210139543	Private: Scripps Research Institute
			CN111991556	Academic: Sun Yat Sen University, Sun Yat Sen University Cancer Center Sun Yat Sen University Affiliated Cancer Hospital
	Coronavirus subunit nanoparticles	Nano-vaccine	CN111603556	Academic: Sun Yat Sen University
Diagnostics	Electrodes and hydrophobic substrate	Sensor and Method for Detecting Target Molecules	US2021255172A1	Academic: Southern Methodist University
	Acoustic sensor	Methods for identifying infectious disease	WO2021221925A1	Company: Aviana molecular Tech LLC
	Carbon-based biosensor	Multiplex biosensor for rapid point-of-care diagnostics	WO2021231948A1	Company: Hememics Biotechnologies Inc
	Fluorescent probe-based biosensor	Fluorescent probe based biosensor and assay for the detection of SARS-CoV-2	US10962529B1	Academic: King Abdulaziz university
	Gold/Silver nanoparticles-aptamer	Optical fiber sensor	CN112730340	Academic: Northwestern University
	Colloidal gold	Immunochromatographic test	CN112485436	Academic: Guilin Electronic Tech University
	Gold nanoparticles	SARS-CoV-2 affinity polypeptide	CN112375754	Academic: Tsinghua University
		Nano plasma resonance	CN111812321	Company: Liangzhun Shanghai Medical Apparatus CO LTD
		Test kit	CN105018644	Government: Jiangsu Provincial Center For Disease Prevention And Control
	Gold magnetic nanoparticles	Nucleic acid detection method	CN112375812	Company: Xian Goldmag Nanobiotech CO LTD
	Magnetic nanoparticles	Anti-SARS-CoV-2 nucleocapsid protein Ig	CN112239501	Company: Dongguan Pengzhi Biotechnology CO LTD
		Test kit	CN112079906	Private:Zhang Ying
	Silver nanoparticles	Silver-complexed therapeutic neutralizing antibody	CN111471105	Academic: Guangxi Medical University
	Oleic acid-modified zinc sulfide nanoparticles	Detection device	CN111579792	Private: Yang Xiaojun
Treatment	Lipid nanoparticles	Nanoparticle Depot For Controlled And Sustained Gene Delivery	US2021330597A1	Academic: New Jersey institute technology
		Anti SARS-CoV-2 drugs	CN112472791	Academic: Fudan University
	Collagen and metal nanoparticles	Collagen as a delivery tool for metal-based anti-viral agents	WO2021229577A1	Company: Collplant LTD
	Chitosan-based nanoparticles	Novochizol	WO2021160667A1	Company: Bosti Trading LTD
	Magnetic nanoparticles	Nano-antibody	CN112500480	Company: Shanghai Novamab Biopharmaceuticals CO LTD
	Polymeric nanoparticles	Alveolar macrophage-like multifunctional nanoparticles	CN112438960	Academic: The Fifth Affiliated Hospital Sun Yat Sen University
	Nanoparticles	gRNA targeting SARS-CoV-2	CN112143731	Company: Guangzhou Reforgene Biological Tech CO LTD

## Can a Fluid and Transversal Network Between Different Skills Favor a Faster and More Effective Management of Health Emergencies Such as the One We Have Been Suffering Worldwide in Recent Years?

In this opinion piece we tried to highlight how, in nanobiotechnology, the intercommunication of skills has allowed technology to design and apply nanoscale solutions for biological applications. Coronavirus prevention, recognition, and handling technologies represent the central weaponries in the fight against Covid diseases. Patent worldwide status mirrors the innovation’s grade in the nanobiotechnological field. According to our opinion, in addition to what has already been designed and implemented, the most ambitious objectives will certainly be achieved with the development of integrated hybrid systems. Recent progresses in nanoscience, smart materials, wireless expertise, and wearable integrated circuit technology allowed Collins and others to engineer a face mask capable of both functioning as an individual protection device and, at the same time, sensing possible viral particles in the wearer’s breath (Nguyen et al., 2021).

We believe that, in the wake of what is already in progress in the nanobiotechnological field with the development of advanced lab on chip and smart nanofeatured devices, it will be possible to develop solutions capable of quickly giving reliable and reproducible results even in personalized medicine, both in diagnostics and in therapy.

We would like to give our opinion on how it could be possible to integrate, on a single microchip, some nano-technologies, nano-features, and nanosystems including thin films and 3D printing for fluidics, separations as molecular and cellular sorting, nucleic acids sequencing, manipulation and genomics, proteomics, or metabolomics for targeted analysis.

Referring to infectious diseases, such as COVID-19, through a simple prick on a finger, using a nanofeatured chip, it could be possible to carry out both the diagnosis and the eventually related post-exposure prophylaxis since testing the presence of the viral RNA or protein simultaneously and estimating how long before the infection started, some therapies, like the one based on monoclonal antibodies, which are efficient only if started before the recommended terms (Taylor et al., 2021), can be administered.

To conclude, although much progress has been made in biotechnological development towards the nanotechnological approach, much still needs to be done in regards to the safety, regulatory, and policy perspective. In our opinion, this could be done by trying to fill the lack of global standardization in the application of robust methods and in the characterization of reference materials through the combination of the needs of consumers/patients and of the skills of scientists, clinics, engineers, jurists, standardization bodies, and companies.

## References

[B1] Allan J. Belz S. Hoeveler A. Hugas M. Okuda H. Patri A. (2021). Regulatory Landscape of Nanotechnology and Nanoplastics from a Global Perspective. Regul. Toxicol. Pharmacol. 122, 104885. 10.1016/j.yrtph.2021.104885 33617940 PMC8121750

[B2] Andersen K. G. Rambaut A. Lipkin W. I. Holmes E. C. Garry R. F. (2020). The Proximal Origin of SARS-CoV-2. Nat. Med. 26 (4), 450–452. 10.1038/s41591-020-0820-9 32284615 PMC7095063

[B3] Carlander D. Skentelbery C. (2021). “EU Regulations and Nanotechnology Innovation,” in Nanotoxicology in Humans and the Environment (Springer), 229–248. 10.1007/978-3-030-79808-6_8

[B4] Casadevall A. Weiss S. R. Imperiale M. J. (2021). Can Science Help Resolve the Controversy on the Origins of the SARS-CoV-2 Pandemic? mBio 12 (4), e01948–01921. 10.1128/mBio.01948-21 PMC8406229 34334001

[B5] Coluccio M. L. Gentile F. Das G. Nicastri A. Perri A. M. Candeloro P. (2015). Detection of Single Amino Acid Mutation in Human Breast Cancer by Disordered Plasmonic Self-Similar Chain. Sci. Adv. 1 (8), e1500487. 10.1126/sciadv.1500487 26601267 PMC4643778

[B6] Contera S. Bernardino de la Serna J. Tetley T. D. (2020). Biotechnology, Nanotechnology and Medicine. Emerg. Top. Life Sci. 4 (6), 551–554. 10.1042/etls20200350 33295610 PMC7752048

[B7] Finnegan M. Duffy E. Morrin A. (2022). The Determination of Skin Surface pH via the Skin Volatile Emission Using Wearable Colorimetric Sensors. Sens. Bio-Sensing Res. 35, 100473. 10.1016/j.sbsr.2022.100473

[B8] Kaloumenou M. Skotadis E. Lagopati N. Efstathopoulos E. Tsoukalas D. (2022). Breath Analysis: A Promising Tool for Disease Diagnosis—The Role of Sensors. Sensors 22 (3), 1238. 10.3390/s22031238 35161984 PMC8840008

[B9] Kirtane A. R. Verma M. Karandikar P. Furin J. Langer R. Traverso G. (2021). Nanotechnology Approaches for Global Infectious Diseases. Nat. Nanotechnol. 16 (4), 369–384. 10.1038/s41565-021-00866-8 33753915

[B10] Nagamine K. Tokito S. (2021). Organic-transistor-based Biosensors Interfaced with Human Skin for Non-invasive Perspiration Analysis. Sensors Actuators B Chem. 349, 130778. 10.1016/j.snb.2021.130778

[B11] Nguyen P. Q. Soenksen L. R. Donghia N. M. Angenent-Mari N. M. de Puig H. Huang A. (2021). Wearable Materials with Embedded Synthetic Biology Sensors for Biomolecule Detection. Nat. Biotechnol. 39 (11), 1366–1374. 10.1038/s41587-021-00950-3 34183860

[B12] Osman E. M. (2019). Environmental and Health Safety Considerations of Nanotechnology, Nano Safety. Biomed. J. Sci. Tech. Res. 19 (4), 14501–14515. 10.26717/bjstr.2019.19.003346

[B13] Park H. Park W. Lee C. H. (2021). Electrochemically Active Materials and Wearable Biosensors for the *In Situ* Analysis of Body Fluids for Human Healthcare. NPG Asia Mater 13 (1), 23. 10.1038/s41427-020-00280-x

[B14] Ruiz‐Hitzky E. Darder M. Wicklein B. Ruiz‐Garcia C. Martín‐Sampedro R. Del Real G. (2020). Nanotechnology Responses to COVID‐19. Adv. Healthc. Mater. 9 (19), 2000979. 10.1002/adhm.202000979 32885616

[B15] Subhan M. A. Subhan T. (2022). “Safety and Global Regulations for Application of Nanomaterials,” in Chapter 5 - Safety and Global Regulations for Application of nanomaterialsNanomaterials Recycling. Editors Rai M. Nguyen T. A. (Elsevier), 83–107. 10.1016/b978-0-323-90982-2.00005-6

[B16] Sung W.-H. Tsao Y.-T. Shen C.-J. Tsai C.-Y. Cheng C.-M. (2021). Small-volume Detection: Platform Developments for Clinically-Relevant Applications. J. Nanobiotechnol 19 (1), 114. 10.1186/s12951-021-00852-1 PMC8058587 33882955

[B17] Susa F. Limongi T. Dumontel B. Vighetto V. Cauda V. (2019). Engineered Extracellular Vesicles as a Reliable Tool in Cancer Nanomedicine. Cancers 11 (12), 1979. 10.3390/cancers11121979 PMC6966613 31835327

[B18] Tavares J. L. Cavalcanti I. D. L. Santos Magalhães N. S. Lira Nogueira M. C. d. B. (2022). Nanotechnology and COVID-19: Quo Vadis? J. Nanopart Res. 24 (3), 62. 10.1007/s11051-022-05452-0 35283662 PMC8901091

[B19] Taylor P. C. Adams A. C. Hufford M. M. de la Torre I. Winthrop K. Gottlieb R. L. (2021). Neutralizing Monoclonal Antibodies for Treatment of COVID-19. Nat. Rev. Immunol. 21 (6), 382–393. 10.1038/s41577-021-00542-x 33875867 PMC8054133

[B20] Weiss C. Carriere M. Fusco L. Capua I. Regla-Nava J. A. Pasquali M. (2020). Toward Nanotechnology-Enabled Approaches against the COVID-19 Pandemic. ACS Nano 14 (6), 6383–6406. 10.1021/acsnano.0c03697 32519842

[B21] Xiao M.-F. Zeng C. Li S.-H. Yuan F.-L. (2022). Applications of Nanomaterials in COVID-19 Pandemic. Rare Mater. 41 (1), 1–13. 10.1007/s12598-021-01789-y PMC8442651 34539132

